# Extraventricular neurocytoma of the sellar region: case report and literature review

**DOI:** 10.1186/s40064-016-2650-2

**Published:** 2016-07-07

**Authors:** Jie Wang, Dong-Lei Song, Li Deng, Shu-Yan Sun, Chun Liu, De-Shan Gong, Yin Wang, Qi-Wu Xu

**Affiliations:** Department of Neurosurgery, The Shanghai Deji Hospital, 378 Gulang Road, Putuo District, Shanghai, China; Department of Pathology, The Peoples’ Hospital of Rizhao, 126 Taian Road, Rizhao, China; Department of Neurosurgery, The Huashan Hospital of Fudan University, 12 Urumchi Road, Shanghai, China

**Keywords:** Sellar region, Extraventricular neurocytoma, Diagnosis, Treatment, Prognosis

## Abstract

**Introduction:**

The extraventricular neurocytoma of the sellar region (EVNSR) is a rare disease, it is difficult to make exact diagnosis of and operate on patients. Retrospectively analysed the clinical manifestations, image features, therapy methods and outcomes among patients with EVNSR, to investigate the epidemiological characteristics, image features, diagnosis, treatment and prognosis.

**Case description:**

A 25-year-old man man with 7-month worsening vision of left eye, was confirmed EVNSR after subtotally resection from the neurosurgical department of Deji hospital.

**Discussion and evaluation:**

Nine cases of EVNSR were reported from this article and elsewhere. Ages of these patients were ranging from 25 to 66 (with an average of 45.67). The male–female ratio was 1–2. All EVNSR patients had visual damage. Images showed the tumors were in the sellar and suprasellar regions. Preoperatively, all patients were misdiagnosed as other diseases: such as pituitary tumor, craniopharyngioma, and meningioma. For tumor removal treatment, five patients received transpterional approach, one received subfrontal approach and three received transnostril–transsphenoidal approach. EVNSR was confirmed by pathological tests. The tumor was completely removed in one patient. During the 12–24 month postoperative follow up period, the recurrence or metastasis of the tumor was found in two patients.

**Conclusions:**

EVNSR is a rare disease. It occurs mostly in middle-aged women. EVNSR is likely to be misdiagnosed as pituitary adenoma preoperatively. The histological examination would help confirm the diagnosis. Using transpterional approach to remove tumor will help the prognosis, especially among patients with normal pituitary function. Both postoperative radiotherapy and long-term follow-up are recommended.

## Introduction

The extraventricular neurocytoma of the sellar region (EVNSR) is a rare disease, with only eigh cases reported in literature up to now. It is difficult to make exact diagnosis of and operate on patients with EVNSR. We report one EVNSR patient receiving radiotherapy on the 5th months after subtotally resection from the neurosurgical department of Deji hospital, Shanghai. We also review the pertinent literatures to investigate the diagnosis, treatment and prognosis of EVNSR.

## Case presentation

**Fig. 1:**
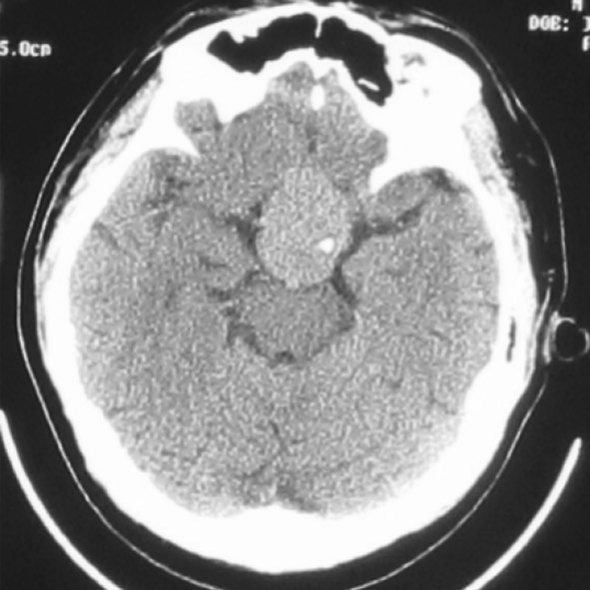
The preoperative cranial CT scan showing a high-density mass in the suprasellar cistern with spot intratumoral calcification

**Fig. 2:**
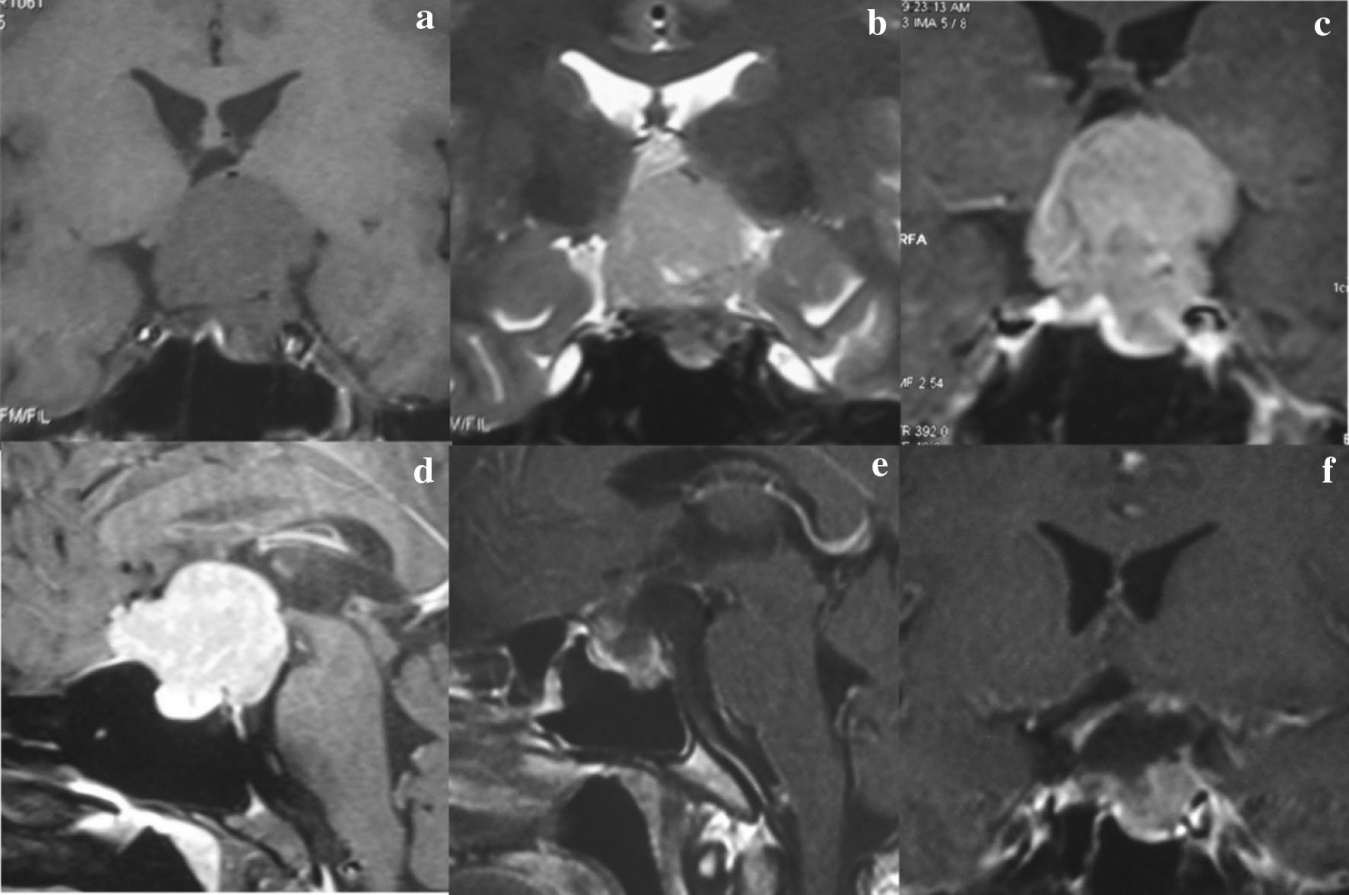
The pre- and post-operative MRI. **a**–**d** Preoperative MRI showing a intrasellar and suprasellar tumor extending to anterior skull base and superior clivus. **e**, **f** The postoperative enhanced MRI showing subtotal tumor removal, with a little tumor remained in the intrasellar region

A 25-year-old man had had worsening vision of left eye since 7 months ago. There was no history of headache, vomiting, polydipsia, polyuria or sexual dysfunction. The case was obese and well-nourished and appeared to be apathic. The visual acuity of right eye was 0.8, while the left eye could only count fingers within 30 cm. There were signs of bitemporal visual field defect, delayed light reflex of the left pupil and slight abduct limitation in left eye. Computed tomography scan showed a roundish homogenous and mildly high density mass in the sellar and suprasellar regions, with clear margin, intra-tumor calcification, but no periphery edema (Fig. 1). The magnetic resonance imaging showed the roundish intrasellar and suprasellar mass of measured 3.9 cm × 3.6 cm × 3.0 cm located slight to the left, which invaded the anterior skull base, cavernous sinus and upper clivus. There were signals of slight hypointense on T1-weighted images (Fig. 2a), slight hyperintense on T2-weighted images (Fig. 2b), moderate nonhomogeneous enhancement (Fig. 2c, d) and the dural tail sign (Fig. 2d), All signals were with clear margin and without periphery edema (Fig. 2a–d). Endocrine tests of the hypothalamic–pituitary axis were normal.

**Fig. 3:**
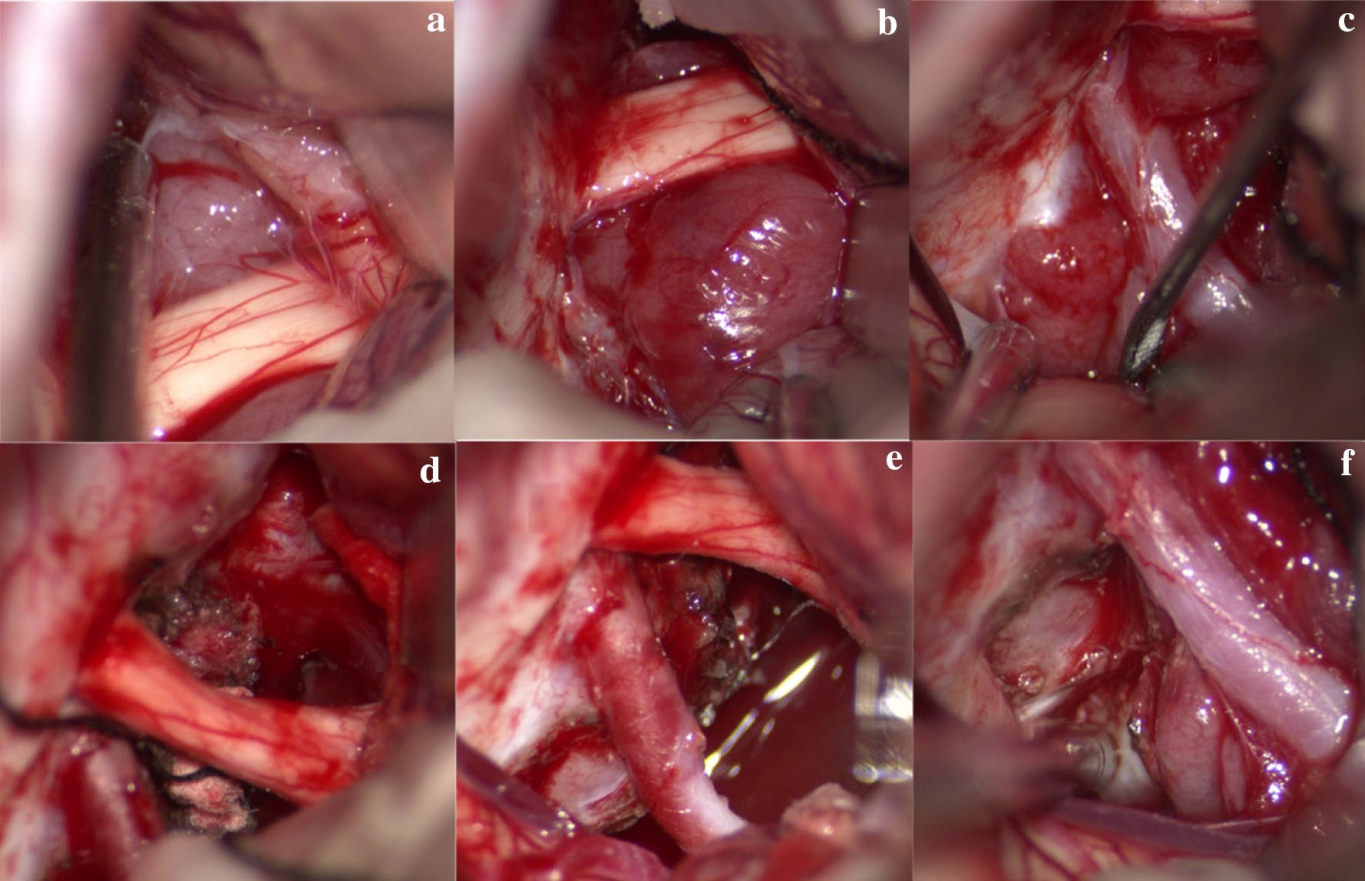
Intraoperative photograph. **a**–**c** The tumor being showed at the interspace I (**a**), interspace II (**b**), interspace III (**c**). **d**–**f** The tumors being resected in the interspace I (**d**), II (**e**), III (**f**)

A left transpterional craniotomy exposed the tumor, which was mainly located in suprasellar region and was partially extended into the intrasellar, anterior skull base, cavernous sinus and upper clivus (Fig. 3a–c). The tumor had soft texture, clear boundary, crimson and hypervascularity. The bilateral optic nerves were compressed severely (Fig. 3a, b). The tumor was then separated along its pseudocapsular margin, and was resected in the interspace I, II, III, respectively (Fig. 3d–f). The tumor in the intrasellar and cavernous sinus was partially removed. The bilateral optic nerves were decompressed well after tumor resection (Fig. 3d, e). The pituitary stalk, bilateral internal carotid arteries, oculomotor nerves and posterior communicating artery were also protected.

**Fig. 4:**
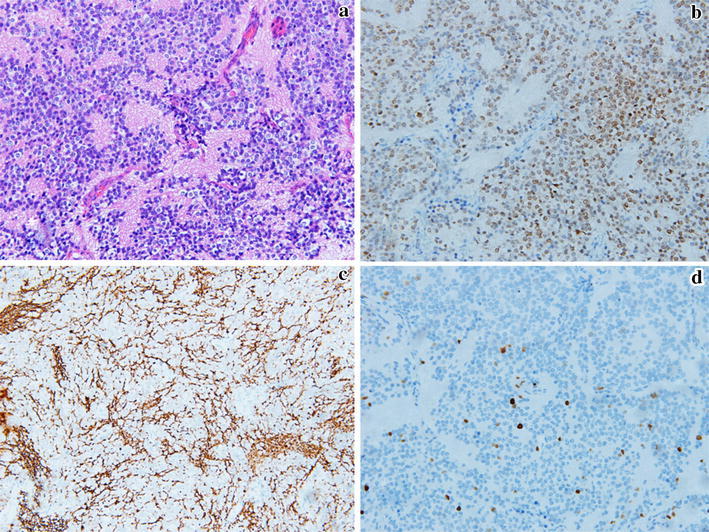
The HE staining and immunohistochemical staining. **a** (HE × 200) Oval well-differentiated tumor cell presenting schistic distribution, with neuropil structure observed in some region. **b**–**d** (Leica BOND-MAX automatic immunohistochemical staining machine × 200) The markers of NeuN (**b**) and Alpha-internexin (**c**) expressing strongly positive, and the Ki67 index being 3 % (**d**)

The resected specimen was grey and soft. Histopathologic examination (HE) staining showed that tumor cells were oval and well-differentiated with schistic distribution. Cytoplasm was sparse with mild acidophilia. Nuclei were oval, without obvious heteromorphosis and mitotic figures. Neuropil structure was observed in some region of the tumor tissues, without necrosis (Fig. 4a). Immunohistochemical staining showed that the neuronal markers such as Synaptophysin, NeuN and Alpha-internexin were highly positive (Fig. 4b–c). Other markers including the glial marker (GFAP), epithelial marker (CK) and anterior pituitary hormones markers (such as GH, PRL, TSH, FSH, LH and ACTH) were negative. The p53 albumen had no mutation. The Ki67 index was around 3 % (Fig. 4d). Pathologic diagnosis was the extraventricular neurocytoma (WHO II).

The patient’s visual acuity improved on the second postoperative day. The patient developed a mild diabetes insipidus but recovered after hypophysin therapy. Cranial MRI showed subtotal tumor removal, with a little tumor remained in intrasellar region (Fig. 2e, f). One week later, the patient developed an epileptic seizure and was treated by oxcarbazepine. The patient was discharged from hospital after 14 days after the surgery.

Three-month postoperative follow-up showed patient’s left visual acuity increased to 0.12 with improvement on vision defect. The anterior pituitary hormone tests were normal. Cranial MRI results were similar as the one taken on the second postoperative day.

## Discussion

Central neurocytoma (CN) is a neuronal and mixed neuronal-glial tumor. It is classified as a WHO II grade tumor (Louis et al. 2007). Central neurocytoma is often called the intraventricular neurocytoma as it originates from septum pellucidum or lateral ventricles, and it grows mostly in the ventricle system. Since the first record by Hassoun in 1982, there has been about 500 cases with CN reported, accounting for 0.10–0.50 % of patients with intracranial tumors. The age of onset is ranging from 40 to 60 years old in 70 % of patients, without gender difference (Choudhari et al. 2009).

Central neurocytoma of extraventricular regions is called extraventricular neurocytoma (EVN). Nearly 100 cases of EVN has been reported, accounting for 0.02–0.10 % of patients with intracranial tumors. EVN is commonly observed among Asians, especially Japanese. The patients’ ages range from 5 to 76 years old with an average of 34 years old. There is no gender difference (Choi et al. 2011; Choudhari et al. 2009). EVN is mostly located at frontal lobe, secondly at parietal lobe. It has also been found at cerebellum, thalamus, brain stem, sellar and spinal cord, as well as regions apart from nervous system such as the retroperitoneal, abdomen, pelvis (Xiao 2013).

Central neurocytoma originated from the sellar region is called extraventricular neurocytoma of the sellar region (EVNSR). EVNSR is a rare disease with only eight cases reported till now. The patients’ age range is from 25 to 66 years old with an average of 45.67 years old. The male–female is 1–2 (Table 1). The major clinical manifestations of EVNSR are hypopsia and vision field defection. Headache, dizziness, and symptoms of the hypertensive intracranial syndrome would also occur. When the tumor erodes pituitary gland or cavernous sinus, endocrinological disorder or impairment of cranial never III or/and VI would occur (Chen et al. 2011). The tumor is round or roundish, sharply defined towards surroundings. The tumor shows slightly high-density on plain CT scan, and has nonhomogeneous enhancing appearance on contrast-enhanced CT scan. Schistic or granular calcification is found over half of the cases, however hemorrhage rarely occurs (Delmaire et al. 2011). The tumor often shows equal or little long T1 signal and equal or long T2 signal. Cyst, calcification and vascular flow voids could be observed when the signal is nonhomogeneous. On the postgadolinium T1-weighted images, the solid part of the tumor shows moderate enhancement (Dominguez-Paez et al. 2011). Since the lack of specificities in clinical manifestations and imaging appearance, EVNSR is often misdiagnosed as other diseases (Table 1). Some literatures recommend MRS examination as an alternative way of differentiation (Kim et al. 2000).

**Table 1:** A concise presentation of the cases with EVNSRs

References	Age/gender	Misdiagnosed as	Clinical features	Images	Treatment	tumor resection extent	Follow-up
Chen et al. (2013)	Female/58 years	Pituitary adenoma	Diplopia, decreased vision of left eye	MRI: A cystic-solid mass in intra/suprasellar region, solid mass revealing iso-T1, and-T2 signal intensity, with obvious enhancement	Transsphenoidal Radiotherapy after operation	Nearly total tumor resection	Left vision improved No recurrence was found in 1 year
Liu et al. (2012)	Male/40 years	Pituitary adenoma	Dizziness blurring of left eye	CT: An irregular hyper-dense mass in intra/suprasellar region	Transpterional Without radiotherapy after operation	Total tumor resection	Left vision improved No recurrence was found in 1.5 year
Pang et al. (2010)	Female/66 years	Pituitary adenoma or Craniopharyngioma	Decreased vision of bilateral eyes, headache, apathy	MRI: An irregular nodular enhanced mass in suprasellar cistern (cavernous sinus invaded)	Transpterional, Radiotherapy after operation	Subtotal tumor resection	Left vision improved and headache relieved. No recurrence was found in 1.25 year
Zhu and Liu (2012)	Female/32 years	Craniopharyngioma	Decreased vision, symptoms of HCP	CT: A slightly hyperdense mass insellar region, with cystic degeneration.	Transsphenoidal Radiotherapy after operation	partial tumor resection	Vision improved and headache relieved. No recurrence was found in 1 year
Wang et al. (2012)	Female/50 years	Pituitary adenoma or Craniopharyngioma	Decreased vision,, bitemporal hemianopia	MRI: A mass insellar region, extended to sphenoid and cavernous sinus CT: Spot calcification	Transsphenoidal Radiotherapy after operation	Partial tumor resection	Vision improved, no recurrence was found in 1 year
Yang et al. (2009)	Female/46 years	Meningioma	Decreased vision of bilateral eyes	MRI: A round mixed signal intensity insellar region, with slight enhancement	Transpterional, radiotherapy after operation	Partial tumor resection	Right vision improved, but tumor recurred in 6 months
Kawaji (2014)	Male/48 years	Pituitary adenoma	Decreased vision	CT: An suprasellar isodense mass MRI: A round suprasellar mix-signal intensity, with obvious enhancement	Bi-subfrontal Radiotherapy after operation	Partial tumor resection	Vision improved, and residual tumor disappeared after radiotherapy, but brain and spinal cord metastasis was found in 6 years later
Wang et al. (2013)	Female/23 years	Pituitary adenoma	Decreased vision, headache	CT: A hyper-density mass in sellar region MRI: intense homogeneous enhancement	Transsphenoidal, radiotherapy after operation	Partial tumor resection	Vision improved and headache relieved
Present case	Male/25 years	Pituitary adenoma or meningioma	Decreased vision in left eye	MRI: A roundish mass in the sellar and suprasellar region	Transpterional, radiotherapy after operation	Subtotal tumor resection	Vision improved

EVNSR is recommended to be removed as much as possible with minimally invasive technique. Clinically, either surgical approach or endoscopic endonasal–transsphenoidal approach will be adopted based on tumor extending direction. The key of treatment is to decompress the optic pathway and to protect the neuro-vascular structure of surgical region. The endoscopic approach may be more effective to protect the optic nerves by causing lesser lesion compared to the craniotomy approach. However, the endoscopic approach may aggravate pituitary malfunction if the tumor evaded to the suprasellar region and remained pituitary tissue. Reconstruction of anterior skull base could be considered via the extended endoscopic approach. But it is not recommended in patients with uncertain diagnosis or in patients with tumor barely eroding the sphenoid sinus.

In our reported case, the preoperative hormone test and image results showed the pituitary gland might remain functional. Instead of the endoscopic endonasal transsphenoidal approach, we decided to use transpterional approach, which we also had more experiences with. Since the tumor slightly predominated at the left side with decreased visual acuity of left eye, we adopted the left transpterional approach to decompress the left optic nerve and to protect the well-functional right one. Microscopically, we successfully resected the tumor in anterior skullbase, the interspace I, II, III, and partial ones in the intrasellar region and the cavernous sinus (Figs. 2, 3). The patient received the radiotherapy (2GY*24, total 48GY) on the fifth postoperative month. The MRI showed obvious tumor reduction on the eighth postoperative month.

The necessity of the postoperative radiotherapy is still controversial. Most scholars believed radiotherapy is effective, especially among cases with partial removal or tumor recurrence. While past cases indicated the total tumor resection to be a more important prognostic factor than postoperative radiotherapy (Table 1), a small number of scholars believed that the overall prognosis is affected by the postoperative radiotherapy (Kim et al. 2013). More patients and longer follow up are needed to make more relevant evaluation for radiotherapy.

Some authors believed EVN had ganglionic and neuroglial differentiation, with higher radio of pathologically atypical features and more postoperative recurrence (Chou et al. 2010). Kane et al. (2012) reported a 44 % 5-year recurrence rate of total eighty-five patients with EVNs and a 14 % 5-year mortality rate. Akshay S concluded in his retrospective analysis that the tumor resection extent was the main prognostic factor, followed by atypical histological features and the patient’s age. The EVNs were more likely to recur in patients at the age of below 18 years old or above 50 years old (Patil et al. 2014). In nearly 100 patients with EVNs reported by Kawaji et al. (2014), five had metastatic focus with one located at cerebellum, three in craniospinal region and one in distant dural. Longer follow-up is needed, especially among patients with residual tumors after operation or atypical tumors (Ki67 > 2 %) and patients at age below 18 years old/above 50 years old. When patient has neurological symptoms and signs hardly explained by tumor recurrence, tumor metastasis should be suspected. In the meantime, the imaging reexamination should be done not only in the surgical regions but also in the regions in which metastasis suspected.

## Abbreviation

EVNSR: extraventricular neurocytoma of sellar region
